# Whipple Disease with Concurrent *Mycobacterium szulgai* Infection: A Rare Diagnostic and Therapeutic Challenge

**DOI:** 10.3390/diagnostics16152315

**Published:** 2026-07-23

**Authors:** Nándor Giba, Kinga Orsolya Dunkel, Eszter Boros, Judit Csomor, Dóra Paróczai, Tamás Lantos, Anita Sejben

**Affiliations:** 1 Department of Pathology, County Hospital Fejér, Szent György Hospital, 8000 Székesfehérvár, Hungary; 2 Department of Internal Medicine, County Hospital Fejér, Szent György Hospital, 8000 Székesfehérvár, Hungary; 3 Department of Pathology and Experimental Cancer Research, Semmelweis University, 1085 Budapest, Hungary; 4 Department of Medical Microbiology, University of Szeged, 6720 Szeged, Hungary; 5 Department of Pulmonology, University of Szeged, 6772 Deszk, Hungary; 6 Department of Medical Physics and Informatics, University of Szeged, 6720 Szeged, Hungary; 7 Department of Pathology, University of Szeged, 6725 Szeged, Hungary

**Keywords:** Whipple disease, *Mycobacterium szulgai*, polymerase chain reaction, Ziehl-Neelsen

## Abstract

Whipple disease is a rare systemic infection caused by *Tropheryma whipplei* that often presents with nonspecific gastrointestinal symptoms and may mimic other disorders. We report the case of a 75-year-old woman with type 2 diabetes mellitus who presented with chronic diarrhoea and a 15 kg unintentional weight loss over 6 months despite preserved appetite. Laboratory investigations revealed iron-deficiency anaemia, hypoproteinaemia, mild inflammatory and liver function abnormalities, and peripheral eosinophilia. Extensive infectious, autoimmune, and gastrointestinal investigations were unrevealing. Abdominal computed tomography demonstrated mesenteric lymphadenopathy, raising suspicion for lymphoma. Histopathological examination of duodenal biopsies revealed numerous foamy macrophages containing periodic acid–Schiff-positive, diastase-resistant granular material, consistent with Whipple disease. Ziehl–Neelsen staining additionally demonstrated acid-fast bacilli, and subsequent investigations confirmed concomitant *Mycobacterium szulgai* infection. The significance of this finding remains uncertain, as it may represent either true coinfection or incidental colonisation. A potential explanation is that *T. whipplei*-induced macrophage dysfunction creates a permissive intracellular niche for nontuberculous mycobacteria, although supporting evidence is lacking. Following antimicrobial therapy, the patient experienced marked clinical improvement with resolution of symptoms and an 8 kg weight gain. This case highlights the diagnostic challenges of Whipple disease and the need to consider concomitant mycobacterial infection when acid-fast organisms are identified.

**Figure 1:**
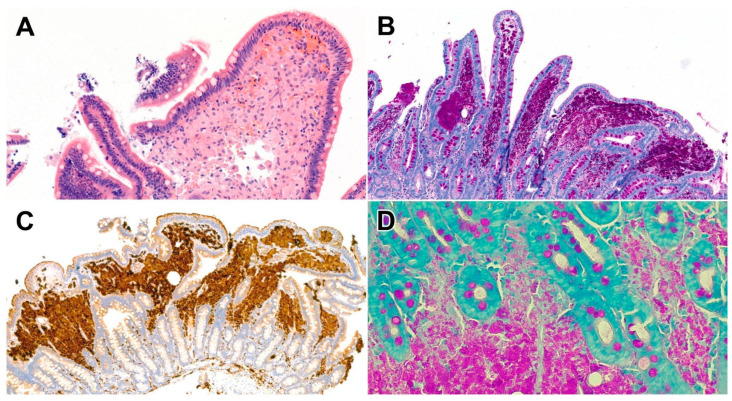
A 75-year-old woman with a known history of type 2 diabetes mellitus was admitted for evaluation of chronic diarrhoea and unintentional weight loss of 15 kg over the preceding 6 months, despite preserved appetite. Initial investigations revealed negative stool cultures and faecal occult blood testing. Laboratory analyses demonstrated iron-deficiency anaemia, hypoproteinaemia, mild elevations in C-reactive protein (CRP) and liver function parameters, as well as peripheral eosinophilia. Screening for human immunodeficiency virus (HIV) infection, including HIV-1/2 antibodies and HIV antigen testing, was negative. Hepatitis serology and parasitological examinations likewise yielded negative results. Evaluation for mycobacterial infection by sputum culture and polymerase chain reaction (PCR) testing for tuberculosis was also negative. In addition, an extensive autoimmune workup was performed, which did not reveal any clinically significant abnormalities. Abdominal ultrasonography suggested impaired intestinal transit with possible small-bowel obstruction. Subsequent abdominal computed tomography (CT) identified mesenteric lymphadenopathy, raising suspicion for an intra-abdominal malignancy, particularly lymphoma. Colonoscopic examination was subsequently performed but revealed no significant pathological findings. Further evaluation with upper gastrointestinal endoscopy demonstrated diffuse oedema, mucosal friability, and a granular appearance of the duodenal mucosa, prompting consideration of coeliac disease and intestinal amyloidosis in the differential diagnosis. Histological examination of the duodenal mucosa demonstrated expansion of the lamina propria by numerous foamy macrophages ((**A**) HE, 40×, (**C**) CD68, 20×) containing abundant periodic acid–Schiff (PAS)-positive, diastase-resistant granular material ((**B**) PAS-D, 40×), corresponding to intracellular *Tropheryma whipplei* organisms. PCR analysis confirmed the presence of *T. whipplei* in the macrophages (LightCycler^®^ HybProbe assay, Roche, Indianapolis, IN, USA). Due to the macrophage accumulation, Ziehl–Neelsen (ZN) staining was carried out that demonstrated positive acid-fast bacilli upon repeat evaluation, as well ((**D**) ZN, 50×). Granulomas were not identified. However, subsequent investigations (GenoType Mycobacterium CM, Hain Lifescience GmbH, Nehren, Germany) confirmed a concomitant infection with *Mycobacterium szulgai* from the paraffin block. Following the initiation of antibiotic therapy, including trimethoprim-sulfamethoxazole (Sumetrolim), ethambutol, and azithromycin-ethambutol-azithromycin, a one-year treatment course was planned for in accordance with ERS guidelines [1]. Recently, the patient experienced marked clinical improvement. The patient did not have symptoms suggestive of pulmonary disease. Chest X-ray and CT imaging were obtained to evaluate the anatomical findings. Sputum culture and PCR testing were negative for tuberculosis. Since then, she has been attending regular gastroenterology follow-up visits. Her condition has improved substantially, and she has gained 8 kg. The patient is retired. Her occupational history includes employment at a television manufacturing facility, where she was involved in television assembly, followed by work in the food service sector as a kitchen employee. Environmental history revealed that her residence underwent professional extermination for bedbug infestation several years prior to presentation. She reported occasional consumption of unwashed fruits harvested directly from her garden. Information regarding personal agricultural activities and previous foreign travel was not available at the time of evaluation; these aspects are planned to be further explored during subsequent follow-up visits. Whipple disease is a rare infectious disease (about 1 case per million people) caused by the Gram-positive bacterium *T. whipplei*, reflecting male predominance and most commonly affecting middle-aged individuals [2,3]. It can present with a wide variety of symptoms, including arthralgia, nausea, vomiting, diarrhoea, and weight loss, while additional nonspecific manifestations may include fever, anaemia, and lymphadenopathy [2,4]. Although *T. whipplei* is ubiquitous in the environment, most infections result in bacterial clearance and seroconversion. In some individuals, persistence leads to asymptomatic carriage, while in rare cases—potentially associated with subtle immune dysfunction—uncontrolled bacterial replication results in classical Whipple disease or localised *T. whipplei* infections [5]. *M. szulgai* is a rare, slow-growing nontuberculous mycobacterium (NTM), accounting for less than 0.2% of NTM isolates worldwide, that most commonly causes a pulmonary disease resembling tuberculosis, particularly in individuals with underlying lung disease or immunocompromising conditions, although extrapulmonary infections can also occur [6,7,8,9]. The interpretation of the ZN positivity in this case warrants careful consideration. One possible explanation is the presence of a true concomitant infection with *M. szulgai*, rather than a diagnostic error or staining artefact. It may be hypothesised that the immunomodulatory effects of *T. whipplei* on host macrophage function create a permissive intracellular environment that facilitates colonisation or infection by NTM. Although this mechanism appears biologically plausible, we were unable to identify published studies specifically addressing an association between *T. whipplei* infection and secondary intracellular mycobacterial colonisation. An alternative interpretation is that the detection of *M. szulgai* represented an incidental finding. NTMs are not infrequently identified in clinical specimens, particularly in elderly or immunocompromised individuals, without necessarily causing clinically significant disease. In such cases, NTM isolation may reflect colonisation rather than active infection. Given that the patient’s gastrointestinal manifestations and subsequent clinical improvement were consistent with Whipple disease, it is possible that the observed symptoms were attributable primarily to *T. whipplei*, while the concomitant mycobacterial finding may have had limited or no contribution to the clinical presentation. Confirmation can be achieved by immunohistochemistry or PCR for *T. whipplei*. Coinfection between *T. whipplei* and mycobacterial species appears to be uncommon. *T. whipplei* is an intracellular pathogen that survives within macrophages and modulates host immune responses, including pathways involved in macrophage activation, polarisation, and apoptosis. These alterations may theoretically impair intracellular bacterial clearance and create a permissive niche for other intracellular pathogens. However, direct evidence linking Whipple disease to increased susceptibility to NTM infection is currently lacking. Rare cases of concomitant *T. whipplei* and *M. tuberculosis* infection have been reported, supporting the biological plausibility of dual infection. In the present case, the identification of acid-fast bacilli and subsequent detection of *M. szulgai* may therefore represent true coinfection, although incidental colonisation cannot be excluded. Furthermore, there is currently no consensus guideline for the management of abdominal NTM infections, and this case underscores the need for the development of evidence-based recommendations in this area.

## Data Availability

The original contributions presented in this study are included in the article. Further inquiries can be directed to the corresponding author.

## Abbreviations

The following abbreviations are used in this manuscript:

CD	Cluster of differentiation
CRP	C-reactive protein
CT	Computed tomography
ERS	European Respiratory Society
HE	Hematoxylin eosin
HIV	Human immunodeficiency virus
NTM	Nontuberculous mycobacteria
PAS	Periodic acid–Schiff
PAS-D	Periodic acid–Schiff–diastase
PCR	Polymerase chain reaction
ZN	Ziehl–Neelsen
